# Anatomical Variations Favouring Antrochoanal Polyp Genesis

**DOI:** 10.7759/cureus.101277

**Published:** 2026-01-11

**Authors:** Sana Ferchichi, Ghada Kharrat, Chiraz Halwani, Senda Turki

**Affiliations:** 1 Department of Otolaryngology, Mohamed Taher Maâmouri University Hospital, Nabeul, TUN; 2 Faculty of Medicine of Tunis, University of Tunis El Manar, Tunis, TUN; 3 Department of Otolaryngology, Military Hospital of Tunis, Tunis, TUN; 4 Department of Otolaryngology, Internal Security Forces Hospital, La Marsa, TUN

**Keywords:** anatomical variant, antrochoanal polyp, computed tomography, etiology, nasal polyp

## Abstract

Background: The antrochoanal polyp (ACP) is a rhinosinusal pseudo-tumour. Its etiopathogenesis is controversial. The antral cyst is thought to be the precursor of ACP. Certain rhinosinusal anatomical variants would favor its transformation into ACP. However, these anatomical variants remain undetermined. The aim of this study was to determine which rhino-sinusal anatomical variants are significantly associated with ACP presence.

Methods: This was a retrospective, descriptive and comparative study of 54 patients operated on for ACP. Clinical and paraclinical data were collected from medical records. A sample of 108 nasal cavities was formed based on the patient list. The sample was divided into two groups: the group of nasal cavities with ACP (ACP+) and the contralateral free group (ACP-). The frequency of each anatomical variant was determined in both groups on the basis of imaging. We compared the frequencies in both groups in order to identify anatomical variants significantly correlated with ACP presence.

Results: The majority of patients were aged 15-30 years, with a range of 13-57 years. The gender distribution showed a male predominance, with 33 male patients and 21 female patients (sex ratio = 1.57). Septal deviation was the most frequent anatomical variant (72.2% of all patients). Among the middle turbinate anatomical variants, concha bullosa was the most represented (33.3% of all patients). A statistically significant association was observed between the ACP+ group and the following anatomical variants (p<0,05): concha bullosa, middle turbinate hypertrophy and hyperpneumatized ethmoid bulla. A statistically significant association was observed between the ACP+ group and the nasal cavity side not obstructed by septal deviation (p=0.016). The mean volume of the maxillary sinus was significantly higher in the ACP+ group (p=0,00). A paradoxical middle turbinate was found in one patient on the ACP+ side and in two patients on the ACP- side. One patient had unciform process anatomical variants: verticalization on the ACP+ side, and pneumatization on the ACP- side. The low number of paradoxical middle turbinate and unciform process anatomical variants did not allow a reliable statistical study.

Conclusion: The nasal septum deviation favours contralateral ACP development. However, concha bullosa, middle turbinate hypertrophy and hyperpneumatized ethmoid bulla cells favour ipsilateral ACP development. We recommend systematically reporting anatomical variations on ACP CT scans, performing wide middle meatotomy during ACP removal, correcting variations that may favor recurrence (septal deviation, concha bullosa) during surgery, and closely monitoring patients with such variations for potential recurrence.

## Introduction

The antrochoanal polyp (ACP), also called Killian polyp, is a rhinosinusal pseudo-tumor that predominantly affects children and young adults [1]. As early as 1891, Zuckerkandl described a “nasal polyp originating from the maxillary sinus, herniating through a wide accessory ostium” [2], but the term “antrochoanal polyp” appeared later, introduced by Professor Gustave Killian, who, in July 1906, described it in his article “The origin of choanal polypi” published in The Lancet [1].

ACP accounts for 4-6% of nasal polyps in the general population and 33% of nasal polyps in pediatric patients [3,4]. It occurs more frequently in men, with a sex ratio of 1.7 men for every woman [5].

ACP originates from a point on one of the walls of the maxillary sinus, mainly the posterior or medial walls [6]. From its site of origin, it passes through the maxillary sinus ostium to reach the ipsilateral nasal cavity toward the choana and may eventually extend into the nasopharynx or even prolapse into the oropharynx [7,8].

The symptoms caused by ACP, dominated by unilateral and progressive nasal obstruction, are nonspecific [9,10]. Diagnosis relies on clinical and radiological assessment, based on the combination of nasal endoscopy and CT scan of the facial bones [11,12]. Treatment is exclusively surgical and well standardized, consisting of endonasal endoscopic polypectomy [13-15].

Thus, various clinical and therapeutic aspects of ACP are currently well established. However, its etiopathogenesis remains insufficiently defined and controversial. The most widely accepted hypothesis implicates the antral cyst (AC) as the precursor of ACP [16,17]. According to this hypothesis, certain physical conditions related to specific rhinosinusal anatomical variants lead to increased pressure within the maxillary sinus. This pressure would cause the AC to herniate into the nasal cavity, mainly through the accessory ostium and more rarely through the primary ostium, giving rise to an ACP.

In the literature, few studies have focused on the pathogenesis of ACP, and even fewer on the anatomical variants that might promote its development [18]. Moreover, the scattered findings that have been published have yielded no definitive conclusions. Yet, understanding the etiopathogenesis of ACP would likely help prevent its occurrence and, above all, its recurrence [19].

Therefore, the objective of our work, through a retrospective study, is to identify the rhinosinusal anatomical variants that are significantly associated with the development of ACP.

## Materials and methods

Study design

In the Otolaryngology Department of Mohamed Taher Maâmouri Hospital in Tunisia, we conducted a retrospective, descriptive, and comparative study. We established a list of patients operated on for ACP who had undergone preoperative CT imaging between January 2010 and December 2022 (13 years). Patients with a history of sinonasal surgery or ACP recurrence were excluded. We recorded the distribution of patients according to age, sex, comorbidities, and presenting symptoms.

Sample collection

Based on this patient list, we constituted a sample consisting of two nasal cavities per patient. The sample was then divided into two groups. The first group included nasal cavities affected by ACP (ACP+). The second group served as the control and consisted of the contralateral, clinically and radiologically unaffected nasal cavities (ACP−).

Data collection

A detailed CT scan review was performed in collaboration with a radiologist. The objective was to evaluate various anatomical variations (nasal septum, turbinates, uncinate process, anterior ethmoidal cells, Haller cells) and to measure the maxillary sinus volume. The latter was calculated blindly on the imaging console by the same observer to minimize observer bias. Measurements were performed manually by contouring the boundaries of the maxillary sinus on successive coronal slices of identical thickness.

Statistical analysis

Our statistical study aimed to investigate the association between certain rhinosinusal anatomical variants and ACP. To this end, we conducted a descriptive analysis of the patients as well as an analytical comparison between the ACP+ and ACP- nasal cavity groups, including both univariate and multivariate analyses. We compared the frequency of each rhinosinusal anatomical variant in the ACP+ group with its frequency in the ACP- group, which served as the control. For qualitative variables, Pearson’s chi-square test was used to compare proportions in independent samples; when the test conditions were not met (expected count less than five), Fisher’s exact test was applied. For quantitative variables with a normal distribution, Student’s t-test was used to compare means between independent samples. A multivariate analysis was then performed to adjust for potential confounding factors and to identify the anatomical variants whose association with the ACP+ group remained statistically significant. The level of statistical significance (p) was set at 0.05. All statistical analyses were carried out using IBM SPSS Statistics for Windows, Version 24 (Released 2016; IBM Corp., Armonk, New York, United States).

## Results

The patient cohort included 54 individuals, yielding a sample of 108 nasal cavities, divided into two groups: ACP+ and ACP−, each comprising 54 nasal cavities.

The majority of patients were aged 15-30 years, with a range of 13-57 years. The gender distribution showed a male predominance, with 33 male patients and 21 female patients (sex ratio = 1.57).

Overall, 53.7% of patients (29 individuals) presented with one or more comorbidities. Among these, nasal hyperreactivity syndrome was the most frequently associated condition, followed by chronic rhinosinusitis (46.3% and 24.1%, respectively). Nasal obstruction was a constant functional sign, observed in all 54 patients, and was unilateral in every case.

**Figure 1:**
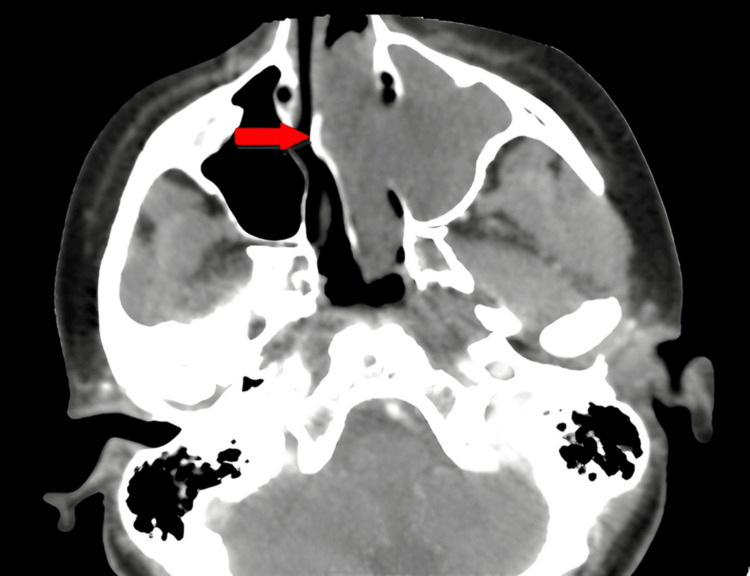
Non-injected axial CT scan: left antrochoanal polyp with septal deviation obstructing the right nasal cavity (red arrow)

Obstruction due to septal deviations was observed in 12 patients (22.2%) in the ACP+ group compared with 27 patients (50%) in the ACP− group. Figure 1 is a representative image from our cohort.

A significant association (p = 0.016) was found between the ACP+ group and the contralateral side of the obstruction caused by septal deviation (p<0.05) (Table 1).

**Table 1:** Summary of comparison of anatomical variations between ACP+ and ACP- groups: Chi² (p<0.05) This table summarizes the anatomical variations in ACP-positive (ACP⁺) and ACP-negative (ACP⁻) groups. Septal deviation, concha bullosa, middle turbinate hypertrophy, paradoxical middle turbinate, inferior turbinate hypertrophy, Haller cells, hyper pneumatized ethmoid bulla (HPEB), agger nasi cells and uncinate process variations were assessed and compared between the two groups. Statistical analysis was performed using the chi-square test (p < 0.05) for all categorical parameters. ACP: Antrochoanal polyp

Anatomical Variation	Group ACP+ N (%)	Group ACP- N (%)	Total N (%)	p-value	χ²
Septal deviation	12 (22.2%)	27 (50%)	39 (72.2%)	0.016	5.87
Concha bullosa	15 (27.8%)	9 (16.7%)	24 (44.5%)	0.011	6.44
Middle turbinate hypertrophy	8 (14.8%)	2 (3.7%)	10 (18.5%)	0.048	3.9
Paradoxical middle turbinate	1 (1.9%)	2 (3.7%)	3 (5.6%)	------	------
Inferior turbinate hypertrophy	14 (25.9%)	16 (29.6%)	30 (55.5%)	0.715	0.13
Haller cells	9 (16.7%)	4 (7.4%)	13 (24.1%)	0.003	8.6
HPEB	13 (24.1%)	8 (14.8%)	21 (38.9%)	0.025	5
Agger nasi cells	7 (12.9%)	8 (14.8%)	15 (27.7%)	0.796	0.07
Unciform process variations	1 (1.9%)	1 (1.9%)	2 (3.7%)	------	-------

The concha bullosa was observed in 15 patients (27.8%) in the ACP+ group and in nine patients (16.7%) in the ACP- group. A middle turbinate hypertrophy was found in eight patients (14.8%) in the ACP+ group, compared to two patients (3.7%) in the ACP- group. The paradoxical middle turbinate was observed in one patient (1.9%) in the ACP+ group and in two patients (3.7%) in the ACP- group. Thus, the ACP+ group had a significant association (p<0.05) with concha bullosa (p = 0.011) and middle turbinate hypertrophy (p = 0.048). The paradoxical middle turbinate cases’ number was insufficient for statistical analysis (Table 1).

The inferior turbinate hypertrophy was observed in 14 patients (25.9%) in the ACP+ group versus 16 patients (29.6%) in the ACP- group, with no statistically significant association (p = 0.715). The Haller cells were found in nine patients (16.7%) in the ACP+ group versus four patients (7.4%) in the ACP- group; this difference was statistically significant (p = 0.03), but this factor was eliminated by the multivariate study (Table 2).

**Table 2:** Summary of multivariate analysis results (Wald test) Summary of multivariate analysis results of anatomical variations. The table presents the results of a multivariate logistic regression analysis using the Wald test. It includes Wald statistics, p-values, standard deviation, and the 95% confidence intervals (lower and upper limits) for each anatomical variable. The anatomical variations analyzed were nasal septal deviation, concha bullosa, middle turbinate hypertrophy, Haller cells, hyper pneumatized ethmoid bulla (HPEB), and mean maxillary sinus volume. Differences were considered statistically significant at p < 0.05

Anatomical Variation	Wald	p	Standard Deviation	Lower Limit	Upper Limit
Nasal septal deviation	8,111	0.004	3.13	1.427	6.865
Concha bullosa	4,137	0.046	2.387	0.95	5.774
Middle turbinate hypertrophy	5,064	0.024	7.913	1.306	47.951
Haller cell	2,050	0.152	2.672	0.696	10.26
HPEB	4,891	0.033	2.179	0.789	6.016
Mean maxillary sinus volume	8,924	0.003	1.915	1.034	3.175

**Figure 2:**
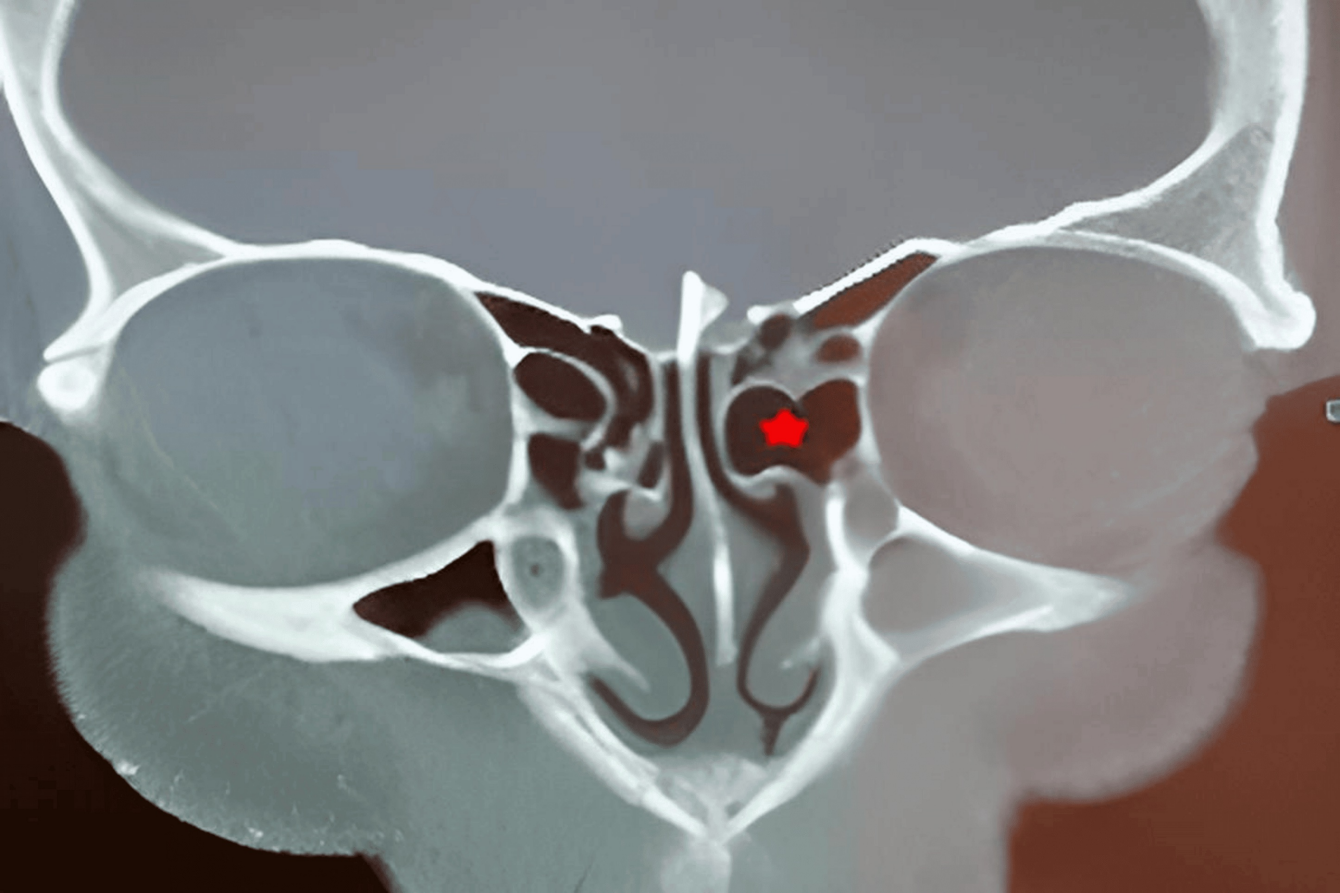
Non-injected coronal section CT scan of the facial mass: left antrochoanal polyp with homolateral hyperpneumatized ethmoid bulla (red star)

The HPEB was present in 13 patients (24.1%) in the ACP+ group versus eight patients (14.8%) in the ACP- group (Figure 2), with a statistically significant association between the ACP+ group and the HPEB presence (p = 0.025) (Table 1).

**Figure 3:**
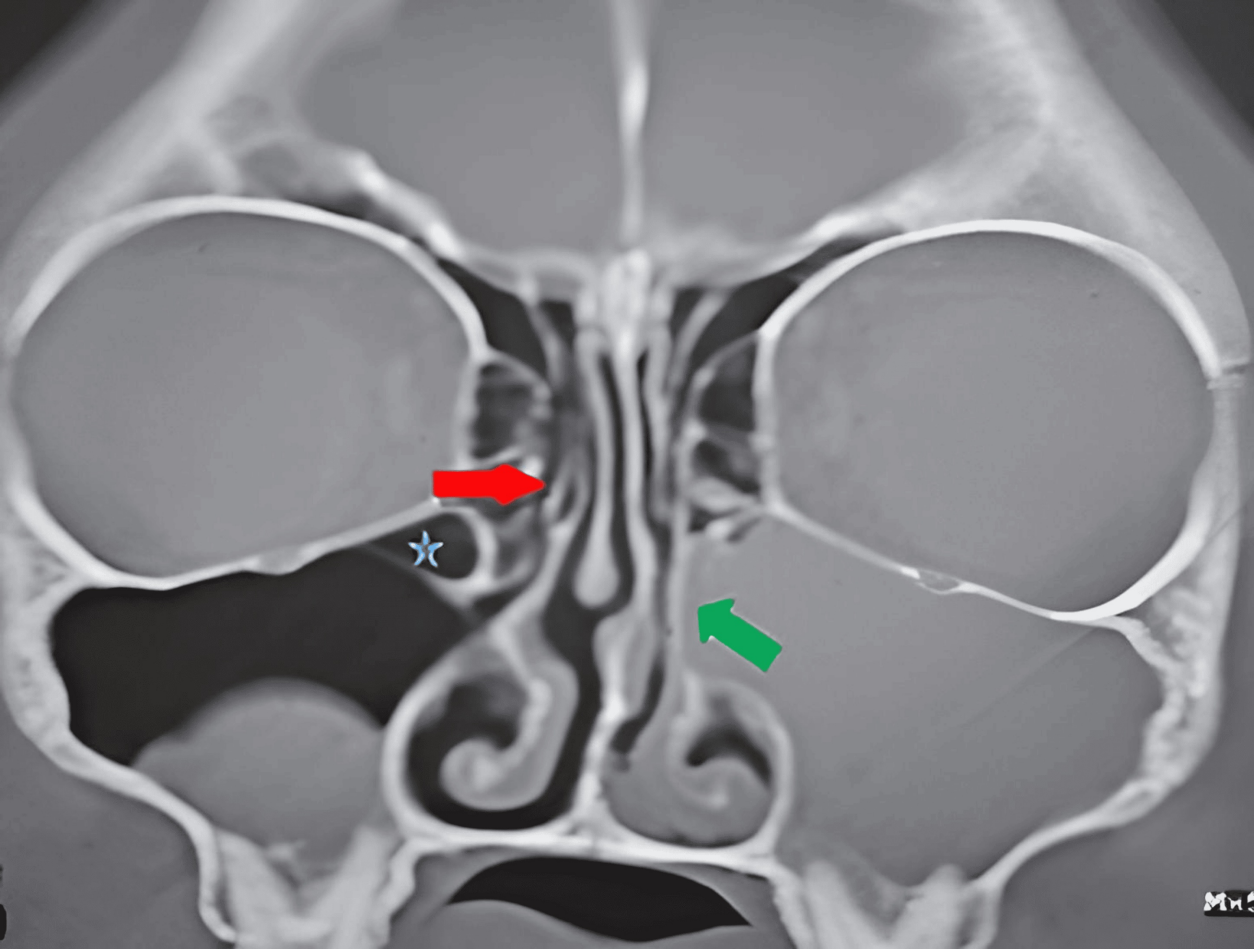
Non-injected coronal section CT scan: left antrochoanal polyp with pneumatized right unciform process (red arrow), right Haller cell (blue star) and left verticalized unciform process (green arrow) Here is a representative image for our cohort showing a non-injected coronal section CT scan: left antrochoanal polyp with pneumatized right unciform process (red arrow), right Haller cell (blue star) and left verticalized unciform process (green arrow).

Agger nasi hypertrophy frequency was observed in seven patients (12.9%) in the ACP+ group versus eight patients (14.8%) in the ACP- group: this difference was not significant. One patient had two anatomical variations of the unciform process: a verticalization on the ACP+ side and a pneumatization on the ACP- side (Figure 3). This number was insufficient for statistical analysis (Table 1).

The mean maxillary sinus volume (MMSV) calculated in the ACP+ group was 18.00 mm³ versus 16.92 mm³ in the ACP- group. The difference was statistically significant (Student’s t-test, p < 0.001). 

## Discussion

Our study had several strengths. We did not include patients with a history of endonasal surgery or with ACP recurrence, as the anatomical structures may be modified and therefore constitute a selection bias. We proceeded with a radiological and not an endoscopic comparison, as some anatomical variations cannot be identified by simple nasal endoscopy (Haller cells, HPEB, Agger Nasi cells). We collaborated with the same radiologist so that the subjective evaluation of some anatomical variations was carried out in the same way in all patients (middle turbinate hypertrophy). Our work included a multivariate statistical study, which adjusted our results by eliminating confounding factors. The originality of our study stems from the fact that the comparison was made on both sides of the nasal cavity in the same patients, so that contralateral sides were used as a control group. This allowed us to eliminate the impact of other factors pertaining to patients that would be common (age, gender, chronic rhinosinusitis and allergic rhinitis). Moreover, the literature review shows that only one other study used the same comparison procedure [9]. Furthermore, this is the third study in the entire literature that has investigated MMSV in patients with ACP [9,20].

Several theories have tried to explain ACP etiopathogenesis. The most widely accepted theory today is that AC is an ACP precursor. In 1988, Berg found that the ACP cystic component and AC had an identical macrostructure, microstructure, and protein distribution in the intracystic fluid [6]. In 2009, Frosini found a perfect histological similarity between the cystic wall of ACP and AC [14]. These pathological findings supported the hypothesis that AC is the precursor of ACP. Moreover, the frequency of AC in patients with ACP is significantly higher than in the general population. Indeed, ACs are present in 8 to 10% of individuals in the general population [21], but this frequency rises to 32.3% in patients with ACP [22]. Anatomically, Berg suggested that chronic inflammation may obstruct the seromucinous glands of the maxillary sinus mucosa, leading to the formation of the AC precursor of ACP [21]. Other authors, such as Piquet, suggest that AC results from stenosis of the lymphatic ducts due to inflammation of the maxillary sinus mucosa [23]. A significant association between ACP and chronic inflammatory conditions (chronic sinusitis, allergic rhinitis, apicodental granulomas) has been noted in multiple studies. Cook's study found allergic rhinitis confirmed by skin tests in 69% of patients with ACP [24]. In addition, Cook noted the coexistence of ACP with chronic rhinitis in over 50% of patients [24].

Our results are consistent with these findings, since we found a history of nasal hyperreactivity syndrome and chronic rhinosinusitis in respectively 46.4% and 24.1% of patients. The AC, whose genesis may be favored by chronic inflammation, is thought to be the ACP precursor [17]. The mechanism responsible for the herniation of a previously silent AC in ACP was explained by Frosini based on Bernoulli's theorem [14]. The same chronic inflammatory factors that promote AC development can also cause mucosal edema and ostio-meatal complex inflammatory stricture. Inflammatory stricture of the maxillary sinus natural ostium as well as complete closure of its accessory ostium obstructed by the AC medial wall can occur simultaneously [7]. Furthermore, according to Bernoulli's theorem, during expiration, the flow of air from the maxillary sinus to the nasal cavity becomes higher at the level of the natural stenotic ostium, creating a pressure drop perpendicular to the wall of the stricture [1]. This results in complete obstruction of the natural ostium during expiration due to the collapse of the walls [21]. Thus, the stricture of the ostio-meatal complex acts as a unidirectional valve, resulting in air trapping inside the maxillary sinus. The simultaneous obstruction of accessory and natural ostia contributes to an increase in intrasinusal pressure. This would lead AC to herniate into the nasal cavity, preferentially through the accessory ostium, and then to ACP formation.

The ACP herniation in the nasal cavity would be favored by any anatomical variation likely to modify the pressure gradient between the nasal cavity, the ostio-meatal complex and the maxillary sinus. Our results confirm the presence of a statistically significant association between the ACP+ group and some anatomical variations (concha bullosa, middle turbinate hypertrophy, HPEB and larger MMSV). Moreover, a statistically significant association was found between the ACP+ group and the contralateral side of the septal deviation obstruction. Our results are close to Başer's findings; however, for the variation "Agger nasi cells", he found a significant association with the nasal cavity side presenting the ACP [9].

All these anatomical variations have in common their location near the middle meatus, and can thus, through the architectural modifications they generate, induce a pressure gradient modification between the maxillary sinus, the ostio-meatal complex, and the nasal cavity concerned. This pressure gradient change is thought to play a promoting role in the ACP herniation through the maxillary sinus accessory ostium. For concha bullosa, middle turbinate hypertrophy, and HPEB, we think that their anatomical proximity to the natural ostium would aggravate the ostio-meatal stenosis and therefore the hyperpressure in the maxillary sinus. For septal deviation, we think that the concavity of the nasal septum on the ACP side widens the nasal cavity and disturbs the airflow and therefore the pressure gradient in the ostiomeatal complex. As for the MMSV, and although it was found to be significantly higher in the ACP+ group, we explain this result as a consequence of ACP direct pressure on the maxillary sinus bone wall rather than a factor promoting ACP genesis.

Due to the limited size of our sample, it was not possible to draw conclusions regarding certain anatomical variations that are usually very rare, such as the paradoxical middle turbinate or anatomical variations of the uncinate process. This limitation has also been noted in other studies [9,14]. In fact, in the literature, the study with the largest sample size (200 patients) was also unable to draw statistically reliable conclusions regarding rare anatomical variations [14].

## Conclusions

Our study identifies a statistically significant association between specific anatomical variations, concha bullosa, middle turbinate hypertrophy, and HPEB, and the ACP+ group, as well as with the side contralateral to septal deviation-related obstruction. These variants, located near the middle meatus, may alter local nasal architecture and modify pressure gradients between the maxillary sinus, the ostiomeatal complex, and the nasal cavity, potentially facilitating ACP herniation through the accessory maxillary ostium, in accordance with Bernoulli’s principle. Although rare anatomical variants could not be analyzed due to limited sample size, this limitation aligns with existing literature.

We recommend systematic reporting of relevant anatomical variations on CT scans, comprehensive surgical management including wide middle meatotomy and correction of contributory variants, and closer postoperative follow-up to reduce recurrence.
